# Sex-specific Doublesex^M ^expression in subsets of *Drosophila *somatic gonad cells

**DOI:** 10.1186/1471-213X-7-113

**Published:** 2007-10-12

**Authors:** Leonie U Hempel, Brian Oliver

**Affiliations:** 1 Laboratory of Cellular and Developmental Biology, NIDDK, National Institutes of Health, 50 South Drive, Bethesda MD 20892 USA

## Abstract

**Background:**

In *Drosophila melanogaster*, a pre-mRNA splicing hierarchy controls sexual identity and ultimately leads to sex-specific Doublesex (DSX) transcription factor isoforms. The male-specific DSX^M ^represses genes involved in female development and activates genes involved in male development. Spatial and temporal control of *dsx *during embryogenesis is not well documented.

**Results:**

Here we show that DSX^M ^is specifically expressed in subsets of male somatic gonad cells during embryogenesis. Following testis formation, germ cells remain in contact with DSX^M^-expressing cells, including hub cells and premeiotic somatic cyst cells that surround germ cells during spermatogenesis in larval and adult testes.

**Conclusion:**

We show that *dsx *is transcriptionally regulated in addition to being regulated at the pre-mRNA splicing level by the sex determination hierarchy. The *dsx *locus is spatially controlled by somatic gonad identity. The continuous expression of DSX^M ^in cells contacting the germline suggests an ongoing short-range influence of the somatic sex determination pathway on germ cell development.

## Background

A regulatory cascade directs all aspects of somatic sexual differentiation in *Drosophila*, including somatic gonad formation [1-3]. This hierarchy is composed of a series of alternative pre-mRNA processing regulators. Diploid flies with two X chromosomes are female (XX:AA) and those with one are male (X:AA). The Sex-lethal (SXL) protein is ubiquitously expressed in early XX:AA embryos and directs female splicing of later appearing *Sxl *and *transformer *(*tra*) pre-mRNA such that functional SXL and TRA proteins are produced only in females. Presence of TRA and the constitutive product of *transformer-2 *(*tra2*) in females lead to female-specific splicing of the *doublesex *(*dsx*) pre-mRNA, which then gives rise to DSX^F ^protein. In X:AA flies, the absence of SXL, and thus TRA, results in male-specific splicing of *dsx *pre-mRNA. Male-specific *dsx *mRNA encodes DSX^M ^protein. Both DSX^F ^and DSX^M ^are zinc-finger transcription factors of the DMRT family. Members of this family play important roles in sex determination in most animals that have been examined to date [4].

The *Drosophila *DSX proteins possess identical N-terminal DNA-binding domains but differ in their C-termini [5-7]. DSX^M ^is thought to repress genes that are involved in female development and activate male differentiation genes while DSX^F ^is thought to do the opposite [8-11]. Although there are a few aspects of sexual dimorphism that are not controlled by DSX, a plethora of phenotypes including elaboration of the abdominal pigmentation, development of the genitalia, sex combs and abdominal neuroblasts, as well as certain aspects of male courtship behavior depend on this important regulator of sexual dimorphism [8,12,13].

The *dsx *locus plays a critical role in both somatic gonad development [14] and specification of germline sexual identity [11,15]. Flies transformed from females to males by constitutive expression of DSX^M ^have testes but very few germ cells. These germ cells can show evidence of either male or female development. However, *dsx *is not required within the germline cells, suggesting that the role of *dsx *in germline development is non-autonomous [16]. Thus, DSX expression is expected in somatic cells that communicate with the germline. Developmental northern blots have shown that there are multiple *dsx *transcripts in larvae and adults [9]. Despite the importance of *dsx *in both somatic gonad and germline development, very little is known about when and where DSX is expressed during gonadogenesis.

Gonad development in *Drosophila *is initiated in the embryo [17]. Germ cells form at the posterior pole of the embryo, divide, are carried into the embryo during gastrulation, migrate through the future gut, and coalesce. Recent work has shown that mesodermal cells from the abdominal region, as well as the germline cells, undergo a well-defined set of migrations to the presumptive gonad and then coalesce into the gonad [18-20]. Additional somatic cells, which express SOX100B, are recruited and maintained in the embryonic testis but not in the ovary; these cells are called male-specific somatic gonadal precursors [21]. Following gonad formation, male germline divisions, regulated by the JAK/STAT pathway, begin [22], whereas there are no divisions of female germ cells at this stage.

In this report we show that the male-specific isoform of *dsx *mRNA is expressed in the embryo. We have developed an antibody that detected the DSX^M ^isoform and show that, in contrast to SXL, which is expressed uniformly throughout the embryo [23,24], DSX^M ^expression was restricted to the initial somatic cells that form the somatic gonad in male embryos. Furthermore, DSX^M ^is expressed in the male-specific somatic gonadal precursors that are later recruited to the gonad and are maintained in male embryos. DSX^M ^is not detected outside the gonad, in the germ cells, or in the late arriving somatic cells that surround the embryonic testis. Male germ cells are in direct contact with DSX^M^-expressing somatic cells through adulthood, as DSX^M ^is specifically expressed in the two somatic cyst cells surrounding developing germline cysts during pre-meiotic spermatogenesis. Only differentiating sperm appear to be unaccompanied by DSX^M^-positive somatic cells.

## Results

### *dsx*^*m *^transcripts in male embryos

**Figure 1:**
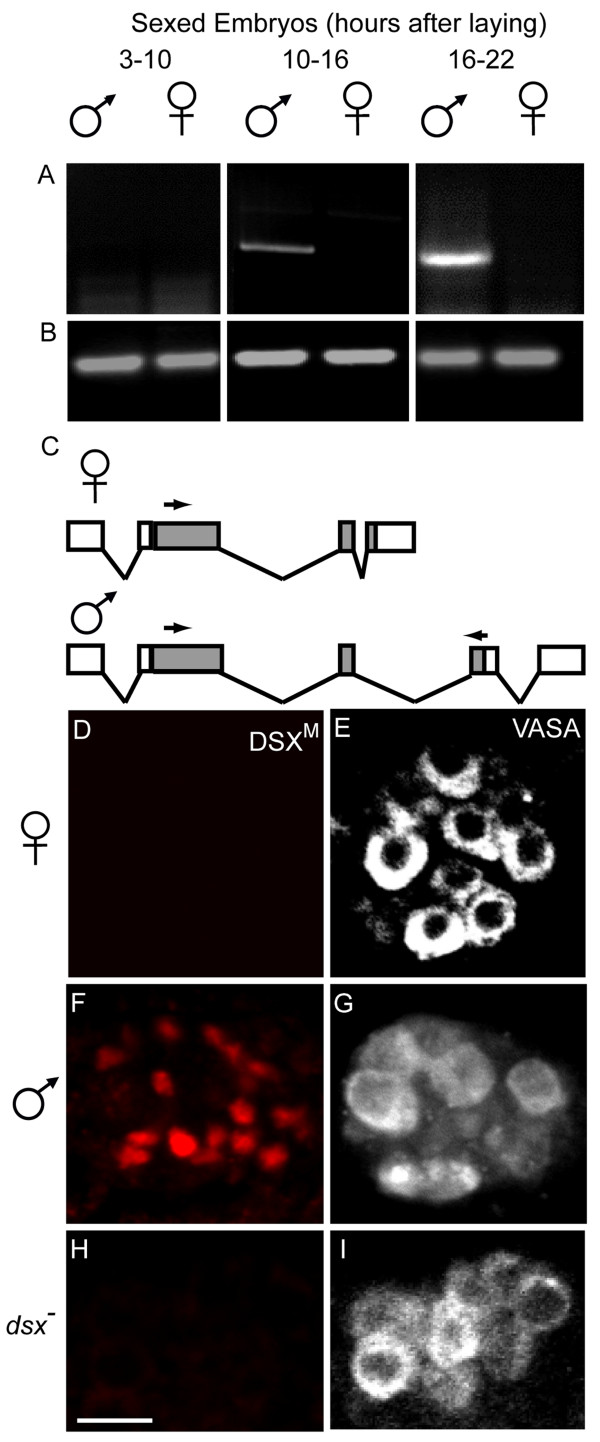
***dsx*^*m *^and DSX^M ^in embryos**. (A) RT-PCR of *dsx*^*m *^from sex sorted embryos of ages 3–10 hours; 10–16 hours and 16–22 hours. (B) *β3-tubulin *amplification control. (C) Cartoon of *dsx *transcripts. The positions of the primers used for the amplification of the male-specific *dsx*^*m *^products are marked (arrows). (D, F, H) Anti-DSX^M ^and (E, G, I) anti-VASA immunofluorescence in (D, E) female, (F, G) male, and (H, I) *dsx*^- ^embryos (*Df(3R)dsx*^15^/*In(3R)dsx*^23^). Images in each row are from the same confocal section of an embryo. Because we used GFP to distinguish homozygous *dsx*^- ^from balancer and heterozygous control embryos, embryos in H, I were not sex sorted. However, we never observed DSX^M ^staining in *dsx*^- ^embryos, 50% of which were male. Secondary antibody for anti-DSX^M ^was biotin-coupled goat anti-rat with tyramide signal amplification, and secondary for anti-VASA was Cy5 goat anti-rabbit. Scale bar = 10 μm.

A systematic survey of embryonic expression patterns has shown that *dsx *transcripts are not maternally deposited and are specifically expressed in the somatic precursors of the gonads just prior to gonad coalescence [25]. However, this global survey did not determine the sex of the embryos or specify the *dsx *mRNA isoform. We sorted male and female embryos bearing a female-specific *Sxl *early promoter [24] attached to eGFP and performed RT-PCR experiments using mRNA from sexed 3–10 hour, 10–16 hour and 16–22 hour old embryos (Fig. 1A–C) to determine if *dsx*^*m *^mRNA is expressed in embryos. *dsx*^*m *^mRNA was not readily detected in 3–10 hour old embryos of either sex, but was easily detected in 10–16 hour and 16–22 hour old male embryos. Sequencing of the RT-PCR products verified that they derived from predicted *dsx*^*m *^transcripts. Thus, RT-PCR experiments showed that *dsx*^*m*^is expressed solely in male embryos.

### DSX^M ^expression in the male embryonic gonad

To further dissect the DSX^M ^expression pattern, we raised polyclonal antisera against a peptide from the male-specific C-terminus of DSX^M^. To determine antibody specificity we performed immuno-labeling experiments focusing on whether: 1) the cell-staining pattern matched the *in situ *hybridization pattern, 2) the signal was male-specific and 3) the signal was absent from male embryos mutant for *dsx*.

The anti-VASA antibody was used to detect germ cells, which served as a guide to follow somatic cells of the gonad. In wild-type male embryos, cells intermingled with VASA-positive germ cells were clearly stained with anti-DSX^M ^during embryonic stage 13 (Fig 1F). Based on position, these were the somatic gonad precursor cells. The DSX^M ^staining was nuclear based on coincident DAPI staining for DNA (additional file 1). Additionally, the pattern of anti-DSX^M ^immunofluorescence coincided with the *dsx*^*m *^transcription pattern detected by *in situ *hybridization previously [25], and in this study (not shown). Finally, we did not observe anti-DSX^M ^immunofluorescence in either female embryos (Fig 1D, E), or in embryos expressing a *dsx *mRNA truncated upstream of the region encoding the epitope used for antibody generation (genotype = *Df(3R)dsx*^15^/*In(3R)dsx*^23^) (Fig 1H, I).

**Figure 2:**
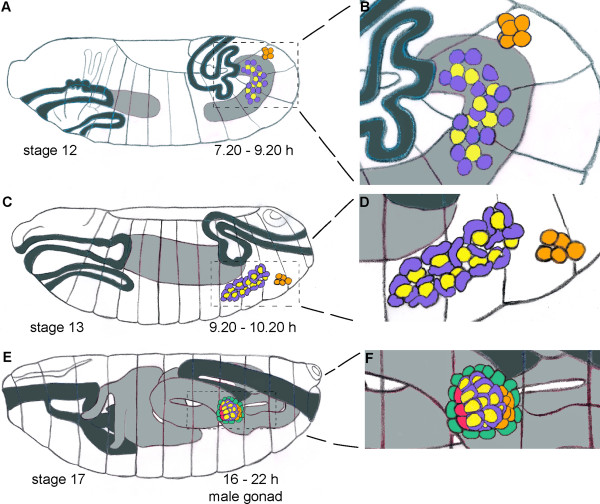
**Gonad development**. Foregut and hindgut (dark-gray); anterior and posterior midgut (light-gray); somatic gonadal precursors (purple); germ cells (yellow); male-specific somatic gonadal precursors (orange); somatic gonadal precursors of the hub (red); and a previously undescribed group of cells (green) are indicated. (A) Stage 12 embryo. (B) Higher magnification view of the outlined area in A. (C) Stage 13 embryo. (D) Higher magnification view of the outlined area in C. (E) Stage 17 male embryo. (F) Higher magnification view of the outlined area in E. Cartoons of embryonic gonad development were adapted from Hartenstein [52]. During gonad formation (A, B) the germ cells and the associated somatic gonad precursors co-migrate towards abdominal segment 5, where they begin to coalesce to form the gonads [53, 54]. During and after gonad coalescence (C, D), the germ cells are intermingled with the somatic gonad cells [41]. Prior to gonad coalescence male-specific somatic gonadal precursor cells, specified in parasegment 13 in both males and females, are located posterior and ventral to non-sex-specific somatic gonad precursor cells. During stage 13 these cells move toward the gonad in both sexes, but only in males do these cells join the posterior of the coalescing gonad. In females these cell die, making the surviving ones "male-specific" [21]. The anterior somatic gonad also becomes sexually dimorphic early during gonad development (E, F). The hub, a cluster of somatic cells required for germline stem cell maintenance in the adult testis, forms anteriorly in the male embryonic gonad [29]. Later in stage 17, we saw another group of cells envelop the embryonic testis (E, F). The identity of these cells is uncertain, but they may be the precursors of the testis sheath [17].

To better determine the identity of DSX^M^-expressing cells, we performed co-immunofluorescence staining experiments with anti-DSX^M ^and antibodies against several somatic gonad precursor markers. We focused on mid- to late embryogenesis when DSX^M ^is strongly expressed and when gonad formation occurs (Fig 2).

**Figure 3:**
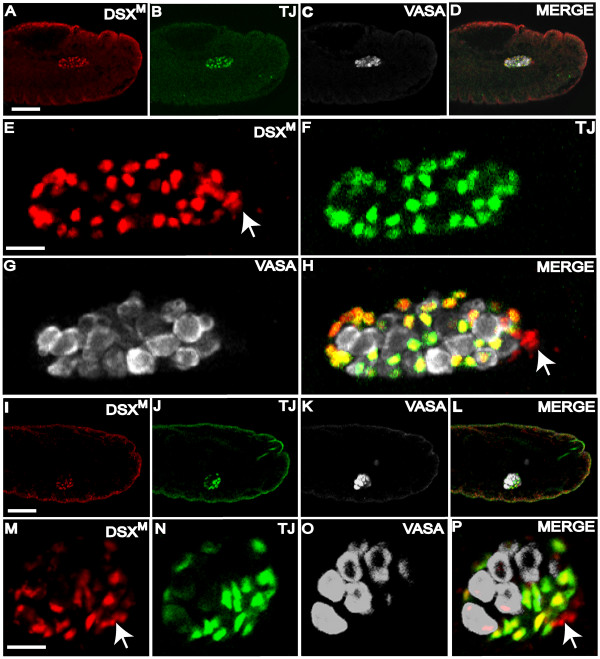
**DSX^M ^and TJ expression in the male somatic gonad**. (A-H) Stage 13 male embryo immunofluorescence using: (A) anti-DSX^M^, (B) anti-TJ, and (C) anti-VASA. (D) Merged images A-C. (E-H) Magnified view of the gonad in A-D. Somatic nuclei expressing DSX^M ^but not TJ are indicated (arrows). (I-P) Stage 15 male embryo immunofluorescence using: (I) anti-DSX^M^, (J) anti-TJ, and (K) anti-VASA. (L) Merged images I-K. (M-P) Magnified view of the gonad in I-L. The scale bars = 50 μm in A-D; I-L and 10 μm in E-H; M-P. Anterior is to the left. Secondary antibodies were: (A, I) biotin-coupled goat anti-rat with TSA, (B, J) Alexa 488 goat anti-guinea pig, and (C, K) Cy5 goat anti-rabbit.

We first ascertained the specific cell type/s that express DSX^M ^by using anti-VASA to detect germ cells and an antibody against the transcription factor Traffic jam (TJ) to detect the somatic gonad precursors [26]. The double labeling with anti-VASA and anti-DSX^M ^indicated that DSX^M ^was not expressed in the germline. Anti-TJ and anti-DSX^M ^staining indicated that DSX^M ^was expressed in somatic cells intermingled with the germline in stage 13 embryos (Fig. 3A–H), stage 15 embryos (Fig. 3I–P), as well as in later embryonic stages.

The overlap in TJ and DSX^M ^staining was consistent with DSX^M ^expression in all somatic gonad precursors in the embryonic testis. However, at the very posterior of the coalescing gonad we detected DSX^M ^expression in cells that did not show TJ staining (Fig. 3E, H and 3M, P arrows), but were clearly assembled into the embryonic testis. TJ is reported to be expressed in all somatic gonad cell precursors [26], but our data suggest that TJ is not expressed in the posterior somatic precursors. Based on position and the confirming experiments outlined below, these DSX^M^-positive and TJ-negative cells were the male-specific somatic gonad cells. This indicates that TJ is not expressed in all somatic gonad cells.

**Figure 4:**
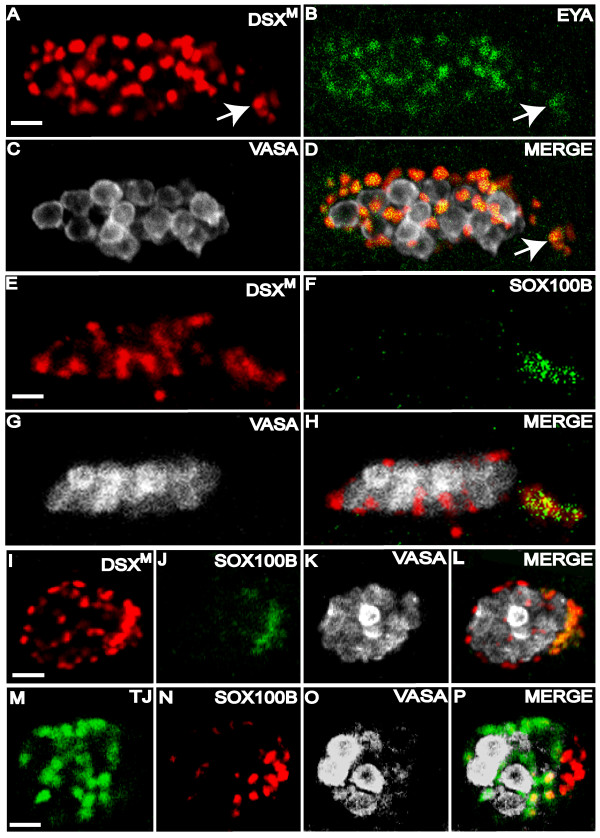
**DSX^M ^but not TJ is expressed in male-specific somatic gonadal precursors**. (A-D) Stage 13 testis immunofluorescence using (A) anti-DSX^M^, (B) anti-EYA, and (C) anti-VASA. (D) Merged images A-C. A DSX^M ^and EYA positive cluster of cell nuclei is located posterior and ventral to the other cells of the somatic gonad (arrows). (E-H) Stage 13 male testis immunofluorescence using (E) anti-DSX^M^, (F) anti-SOX100B and (G) anti-VASA. (H) Merged images E-G. (I-L) Stage 15 testis immunofluorescence using (I) anti-DSX^M^, (J) anti-SOX100B, and (K) anti-VASA antibody. (L) Merged images I-K. (M-P) Stage 15 testis immunofluorescence using (M) anti-TJ, (N) anti-SOX100B and (O) anti-VASA. (P) Merged images M-O. The scale bars = 10 μm. Anterior is to the left. Secondary antibodies were: (A, E, I) biotin-coupled goat anti-rat and TSA, (B) Alexa 488 goat anti-mouse, (C) Cy5 goat anti-rabbit), (F, J) Alexa 488 goat anti-rabbit, (G, K, O) Alexa 647 goat anti-chicken, (M) Alexa 488 goat anti-guinea pig, (N) biotin-coupled goat anti-rabbit and TSA.

To further investigate DSX^M ^expression in the somatic gonad we used anti-Eyes absent (EYA). EYA is a transcription factor, which is also expressed in the somatic gonad [27]. EYA was found in the entire somatic gonad including the cluster of posterior TJ-negative somatic gonad cells. Staining for EYA and DSX^M ^revealed co-expression at the cellular level throughout the entire somatic gonad of stage 13 and older embryos (Fig. 4A–D). These data suggest that a small population of somatic gonad cells expresses DSX^M ^and EYA, but not TJ.

To ascertain the identity of the posterior most cells of the somatic gonad more directly, we applied anti-DSX^M ^in combination with anti-SOX100B, because the male-specific somatic gonad precursor cells are reported to be the only gonad cells expressing SOX100B [28]. Anti-DSX^M ^and anti-SOX100B co-immunofluorescence revealed DSX^M ^expression in male-specific somatic gonad precursors (Fig. 4E–L). Absence of TJ in a subset of SOX100B-expressing cells was confirmed by counter-staining with anti-SOX100B (Fig. 4M–P). There were clearly cells expressing SOX100B, but not TJ. A few cells appeared to express both SOX100B and TJ. Taken together, these data indicate that DSX^M ^is expressed in all somatic gonad cells expressing EYA and either TJ or SOX100B.

**Figure 5:**
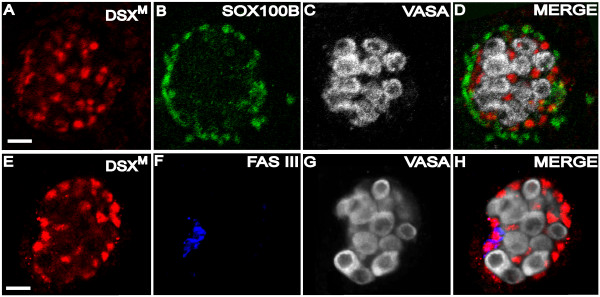
**DSX^M ^is not expressed in all somatic testis cells**. (A-D) stage 17 testis immunofluorescence using (A) anti-DSX^M^, (B) anti-SOX100B, and (C) anti-VASA. (D) Merged images A-C. (E-H) stage 17 testis immunofluorescence (imaged in a focal plane with the hub) using (E) anti-DSX^M^, (F) anti-FAS III, and (G) anti-VASA. (H) Merged images E-G. The scale bars = 10 μm. Secondary antibodies were: (A, E) biotin-coupled goat anti-rat and TSA, (B) Alexa 488 goat anti-rabbit, (C) Alexa 647 goat anti-chicken, (F) Alexa 647 goat anti-mouse, and (G) Cy5 goat anti-rabbit.

Interestingly, SOX100B was also expressed in another population of cells that wrapped around the gonad in stage 17 embryos (Fig. 5A–D). These might have been the precursors of the testis sheath [17], but we did not investigate the fate or function of these cells in this study. We did not observe DSX^M ^expression in these SOX100B-positive cells surrounding the embryonic testis (Fig. 5A–D).

The testis hub is part of the niche and required to maintain male germline stem cell identity. The somatic hub cells of the male gonad can be identified in stage 17 embryonic testes as an anterior cluster of somatic cells expressing the cell adhesion molecule Fasciclin III (FAS III) [29]. In anti-DSX^M ^and anti-FAS III co-immunofluorescence experiments, we observed strong DSX^M ^expression in hub cells that were outlined by anti-FAS III labeling (Fig. 5E–H). Taken together, our data indicate that DSX^M ^is expressed in all the somatic cells of the embryonic testis identified with somatic gonad markers, except the SOX100B-positive cells that surround the embryonic testis at stage 17.

### DSX^M ^is expressed during spermatogenesis in cyst cells prior to meiosis

In contrast to many other cell types, the germline stem cells continue to divide in adults in order to produce gametes. If germline sexual identity is irreversibly determined during embryogenesis, there may be no need for post-embryonic somatic DSX^M ^expression. However, if sex determination or maintenance of sexual identity is an ongoing process, then germline sexual identity might require continued expression of DSX. We readily detected *dsx*^*m *^transcripts by RT-PCR with RNA from adult testes (not shown). To explore the cellular expression pattern of DSX^M ^in testes of larvae and adult flies, we performed whole-mount antibody immunofluorescence staining experiments.

**Figure 6:**
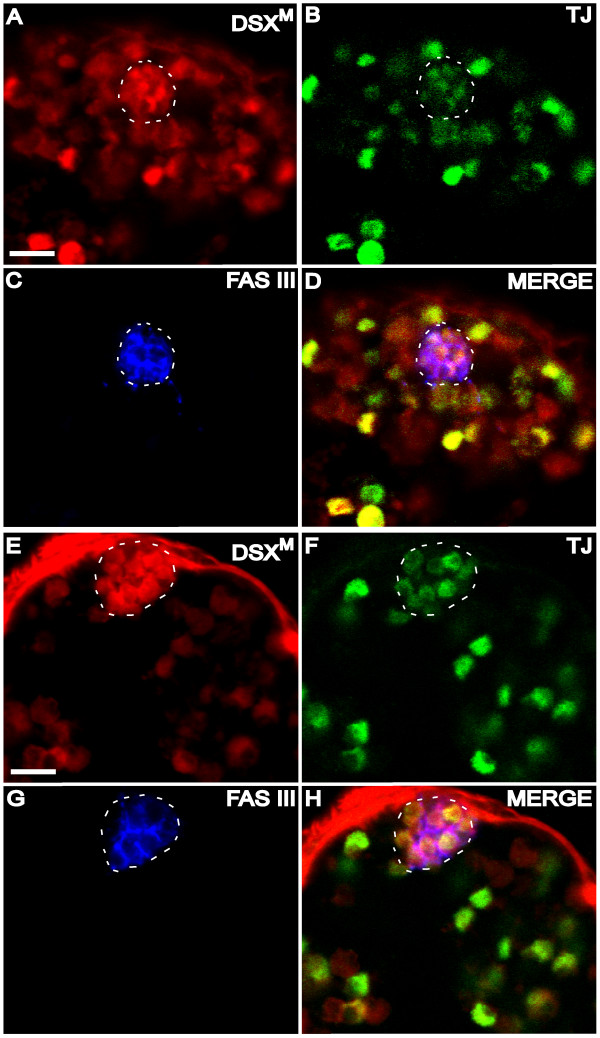
**DSX^M ^in hub cells**. (A-D) Larval (3rd instar) testis immunofluorescence using (A) anti-DSX^M^, (B) anti-TJ, and (C) anti-FAS III. (D) Merged images A-C. (E-F) Adult testis immunofluorescence using (E) anti-DSX^M^, (F) anti-TJ, and (G) anti-FAS III. (H) Merged images E-G. The hub is outlined (white dashes). The scale bars = 10 μm. Anterior is up and out of the plane toward the viewer (the outlined hub is most anterior). Secondary antibodies were: (A, E) biotin-coupled goat anti-rat and TSA, (B, F) Alexa 488 goat anti-guinea pig, and (C, G) Alexa 647 goat anti-mouse.

Male germline stem cells in close contact with the hub undergo asymmetric divisions to regenerate the stem cell population and produce the cells that develop into sperm [30]. Cells remaining at the hub are the stem cells. We observed DSX^M ^expression in the somatic hub cells of larval (Fig. 6A–D) and adult testes (Fig. 6E–H), where it also co-localized with TJ. These data suggest that male germline stem cells are always in contact with DSX^M^-expressing cells.

**Figure 7:**
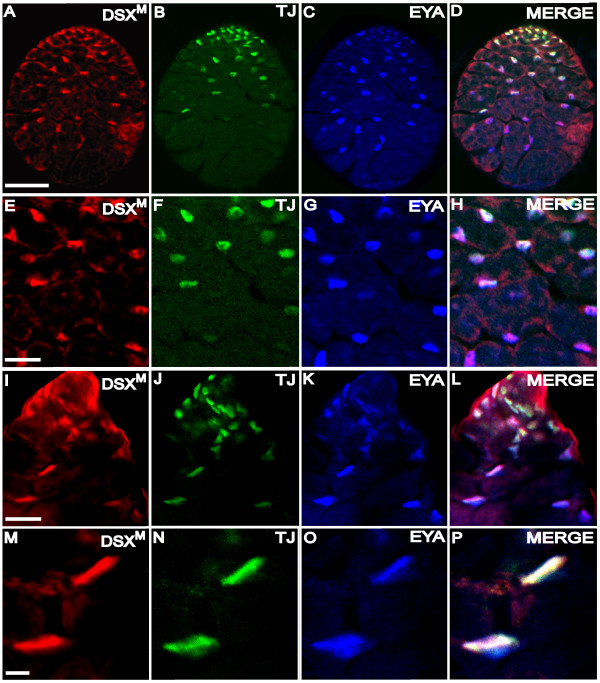
**DSX^M ^expression during spermatogenesis**. (A-D) Larval testis immunofluorescence using (A) anti-DSX^M^, (B) anti-TJ, and (C) anti-EYA. (D) Merged images A-C. (E-H) Higher magnification view of A-D. (I-L) Adult apical testis immunofluorescence using (I) anti-DSX^M^, (J) anti-TJ and (K) anti-EYA. (L) Merged images I-K. (M-P) Higher magnification view of I-L. The scale bar = 50 μm in A-D, 20 μm in E-L, and 5 μm in M-P. Anterior is up. Secondary antibodies were: (A, I) biotin-coupled goat anti-rat and TSA, (B, J) Alexa 488 goat anti-guinea pig, (C, K) Alexa 647 goat anti-mouse.

Germline cells leaving the niche divide four times to produce 16-cell cysts. Germline cysts are enveloped by two somatic cyst cells, which become flattened as the germline cysts enlarge 20-fold in volume in preparation for meiosis. These engorged cells are highly transcriptionally active. Sperm differentiation occurs postmeiotically under translational control [30]. The transcription factor TJ is expressed in the cyst progenitor cells at the apex and in early cyst cells enveloping the dividing germ cells and then fades in later cysts [26]. EYA expression becomes stronger during this progression, such that the two patterns are partially complementary [31]. Co-immunofluorescence with anti-DSX^M^, anti-TJ and anti-EYA revealed that there was overlapping expression of the three proteins in these somatic cyst cells (Fig. 7). Anti-DSX^M ^staining of larval and adult testes revealed expression in early cyst cells as evidenced by position within the gonad and the overlap with TJ. When TJ expression faded, DSX^M ^expression persisted in late cyst cells and co-localized with EYA (Fig. 7A–D and 7E–H). Interestingly, anti-DSX^M ^staining was never observed in cyst cells surrounding transcriptionally quiescent post-meiotic stages (not shown). These data are consistent with the idea that the determination or maintenance of germ cell sexual identity requires ongoing DSX^M ^expression until the end of spermatogenic transcription at meiosis, but is no required once germline transcription ceases.

### DSX^M ^expression does not require a germline, EYA, or TJ

**Figure 8:**
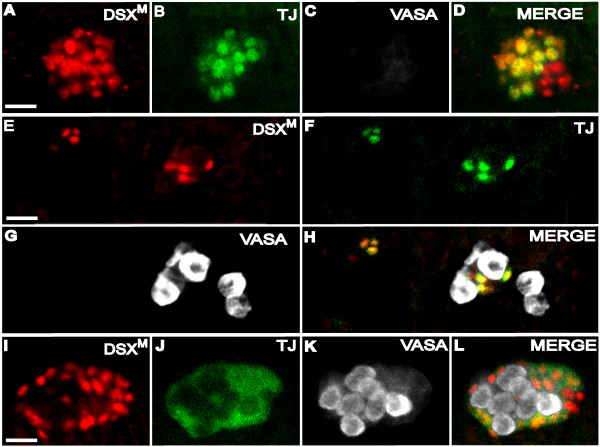
**DSX^M ^expression does not require germ cells nor EYA or TJ**. (A-D) Agametic testis of stage 15 males from *gs(1)N26 *mothers. Immunofluorescence using (A) anti-DSX^M^, (B) anti-TJ, and (C) anti-VASA. (D) Merged images A-C. (E-H) Isolated somatic gonadal precursors and germ cells formed in homozygous *eya *mutants. Immunofluorescence using (E) anti-DSX^M^, (F) anti-TJ, and (G) anti-VASA. (H) Merged images E-G. (I-L) Expression in *tj*^*eo *^mutant testis revealed with immunofluorescence using (I) anti-DSX^M^, (J) anti-TJ, and (K) anti-VASA. (L) Merged images I-K. Expression of the non-functional truncated TJ protein in *tj*^*eo *^mutant embryos is diffuse within the somatic gonadal precursors (J). The scale bars = 10 μm. Anterior is to the left. Secondary antibodies were: (A, E, I) biotin-coupled goat anti-rat and TSA, (B) Alexa 647 goat anti-guinea pig, (C) Alexa 488 goat anti-rabbit, (F, J) Alexa 488 goat anti-guinea pig, and (G, K) Cy5 goat anti-rabbit.

Testis formation depends on the collaboration of germ cells and distinct somatic cell types. However, somatic gonad formation is independent of germ cells [17,32]. Therefore, we predicted that functional DSX^M ^expression should not be dependent on a germline. To investigate whether germ cells were necessary for DSX^M ^expression in somatic gonadal precursor cells, we examined embryos lacking germ cells due to the maternal grandchildless mutation *gs(l)N26 *[33]. Unsurprisingly, anti-DSX^M ^staining was readily detectable in germlineless embryonic testes (Fig. 8A–D) indicating that the germline is not required to induce or maintain DSX^M ^expression.

The *eya *gene was a candidate regulator of *dsx *expression as EYA was always expressed in DSX^M^-positive gonad cells, and preceded DSX^M ^expression temporally in the embryo (EYA was strongly expressed in somatic gonadal precursor cells of stage 12 embryos, when we did not readily detect DSX^M ^expression). We therefore determined whether DSX^M ^expression required EYA function. This experiment was complicated by the fact that EYA is essential for gonadogenesis. In late embryonic stages, *eya *mutant embryos have germ cells that are scattered throughout posterior regions and only a few somatic gonad precursor cells develop. The few somatic gonad precursors do not coalesce into a gonad, rather they form clumps with associated germ cells [27]. In homozygous *eya *mutant male embryos, we were able to detect DSX^M ^expression in the sporadically formed somatic gonad precursor cells (Fig. 8E–H). These sporadically formed cells also expressed TJ. Expression of DSX^M ^and TJ in these rarely formed somatic gonad precursor cells suggests that *eya *is not obligatory for expression of TJ or DSX^M ^in the embryonic testis. However, it should be noted that there are very few TJ- and DSX^M^-positive cells in *eya *mutants. Those somatic gonad cells that escape may not be representative.

TJ was expressed in many DSX^M^-positive somatic cells and was therefore a potential regulator of *dsx*. We examined *tj*^*eo*2 ^male embryos to determine if DSX^M ^expression depends upon TJ. The *tj*^*eo*2 ^allele [26] encodes a truncated protein lacking the two DNA-binding domains, a putative bipartite nuclear localization signal, and the leucine-zipper domain. The allele behaves as a genetic null. In gonads of *tj *mutant embryos, germline divisions during late embryogenesis are hindered. Additionally, somatic gonad cells do not intermingle with germ cells and remain at the gonad periphery [26]. In homozygous *tj *mutant male embryos, we detected DSX^M ^expression in all the somatic gonad cells (Fig. 8I–L). Thus, DSX^M ^expression in somatic gonad cells is not dependent upon the activity of TJ.

## Discussion

### Spatial and sexual regulation of DSX^M^

**Figure 9:**
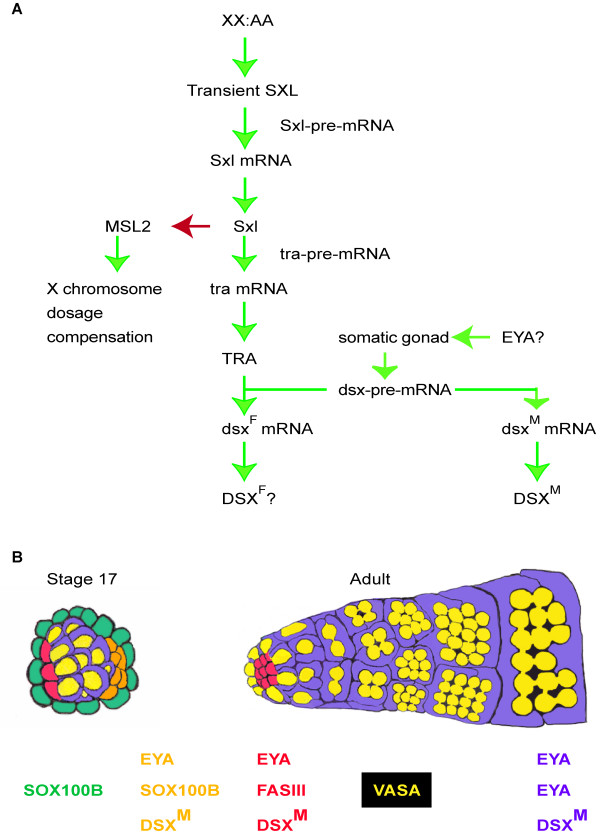
**DSX^M ^regulation**. DSX^M ^is regulated by the intersection of the sex-determination alternative pre-mRNA splicing hierarchy and spatial/temporal regulation (A). Positive (green arrows) and negative interactions (red) are indicated. See text for details. Cellular markers are differentially expressed in somatic cell types of the male gonad during embryonic and adult stages (B). Cell cartoons and expression indicators are color-coded. Germ cells (yellow), somatic gonad precursors and cyst-cells (purple), male-specific somatic gonadal precursors (orange), hub cells (red), and a novel layer of embryonic testis cells (green) are shown.

The pre-mRNA splicing cascade regulating somatic sexual development has been well studied (Fig 9A). The X-chromosome number is read by the uniform expression of X chromosome transcription factors, which uniformly and transiently activate *Sxl *expression in XX:AA flies prior to general activation of the zygotic genome [3]. This transient expression of the SXL splicing factor results in an autoregulatory loop when *Sxl *pre-mRNA is produced from a ubiquitous promoter active later in development. In X:AA flies, SXL is not present and dosage compensation complexes form in all X:AA cells to increase X chromosome gene expression [34]. Addressing chromosome dose imbalance should be important for all cells. The uniform and early expression of SXL to prevent over-expression of X chromosome genes, via inhibition of male-specific-lethal-2 (MSL2) in females, is therefore quite logical. The absence of SXL also ultimately results in DSX^M ^production. As many aspects of sexual dimorphism in the soma require DSX function [8,14,35-38], there is no *a priori *requirement for tissue-specific expression of *dsx*.

Here we show that DSX^M ^expression in male embryos is restricted to somatic gonadal cells that form several hours after SXL expression is initiated. Consequently, in addition to the sex determination hierarchy, it is likely that DSX^M ^expression depends on additional and yet unknown activators expressed in embryonic somatic gonadal precursor cells or repressors in non-gonadal somatic cells. It is also likely that DSX^F ^is expressed in the corresponding somatic gonad in females, but we have not been able to generate useful antibodies against the predicted female isoform, or the region common to DSX^M ^and DSX^F^. Expression of *dsx *in subsets of the nervous system [39] raises the possibility that transcriptional deployment of *dsx *pre-mRNAs in female or male pre-mRNA splicing environments is a common theme.

### Defining subsets of somatic testis cells

Two main cell types compose the embryonic *Drosophila *gonad: germ cells and somatic gonad cells [19]. Our results suggest that each somatic gonadal cell of a male stage 13 embryo has a male identity before overt morphological testis development is evident. By stage 17, embryonic gonads are clearly sexually dimorphic (Fig 9B). The hub, a cluster of specialized somatic cells required for germline stem cell maintenance, forms anteriorly in stage 17 testes [29]; these hub cells express DSX^M^. We have also seen a monolayer of cells around the testis during stage 17, possibly the testis sheath precursors. These late additions to the embryonic testis do not express DSX^M ^but do express SOX100B.

### Somatic DSX^M ^and the germline

Although DSX^M ^is required to promote male development and to repress female development in several somatic tissues, the role of DSX^M ^activity in embryonic testis morphogenesis is unclear as *dsx *mutants are strikingly similar to wildtype males [21]. However, it is clear that DSX^M ^regulates the expression of the STAT92E transcription factor in male embryonic germ cells [22]. Additionally, DSX^M ^results in male-specific splicing of *Sxl *pre-mRNA in adult germ cells [15]. Perhaps the major role of somatic DSX^M ^in the embryo is regulation of the germline gene expression program. The non-autonomous role of DSX^M ^is not restricted to germline development, as DSX^M ^also non-autonomously regulates the post-embryonic recruitment of mesodermal cells to the male genital disc [40].

DSX^M^-expressing cells are in intimate contact with the germline, which may be important to enable the somatic sex determination pathway to influence germ cell development in the embryonic gonad. Somatic gonadal precursors undergo striking shape changes as the gonad coalesces, producing thin cellular extensions that surround the round germ cells at stage 13 [41]. DSX^M ^expression becomes apparent at this stage.

There is also contact between germline and DSX^M^-positive somatic cells in post-embryonic stages. We found that DSX^M ^is expressed in testes of larvae and adult flies in the somatic cyst cells enveloping all premeiotic germ cell cysts as well as in the hub. DSX^M ^expression was never observed in cyst cells enclosing postmeiotic germ cells, which have completed the transcriptional program required for sperm differentiation [42,43]. Thus, DSX^M ^appears to be expressed in all the somatic cells that are closely associated with the germ cells from gonad formation until the transcriptional program of spermatogenesis is complete.

## Methods

### Flies

All flies were raised and maintained on standard cornmeal media (Tucson Drosophila stock center, Tucson, AZ) at 25°C. A fly strain bearing a construct containing the early *Sex lethal *promoter, *SxlP*_*E*_, [24] upstream of the coding sequence for eGFP on chromosome 3 *(Sxl-GFP-3) *was used for sexing embryos [44]. Only female embryos of this line express eGFP. Dechorionated embryos were sex sorted by distinguishing fluorescent versus non-fluorescent embryos using the Copas Select sorter (UNION Biometrica, Holliston, MA). 60 embryos from each collection were set aside and allowed to mature to score the sexing efficiency. Only collections showing 100% sexing fidelity were used in experiments. The following mutant alleles were used for analyses: *gs(l)N26 *[45]; *Df(3R)dsx*^15 ^and *In(3R)dsx*^23 ^[46]*; eya*^*cli-IIE*^and *eya*^*cli-DI *^[47] and *tj*^*eo*2 ^[26].

### RT-PCR

Total RNA was prepared using TRIzol reagent (Invitrogen, Carlsbad, CA) from 3–10, 10–16, 16–22 hour old, sex sorted embryos as well as from testes of male adult flies. Preparation of polyA^+^-mRNA from all samples was done using the Oligotex mRNA Mini Kit (Qiagen, Valencia, CA). RT-PCR was performed using the OneStep RT-PCR Kit (Qiagen, Valencia, CA). A pair of primers (RT-sense 5'-CGCGCACCACGTCCACATGGCAGCTG-3'; male-antisense 5'-CTCTGGAGTCGGTGGACAAATCTGTGTG-3') flanking two introns was used to amplify an 801 bp cDNA fragment from the *dsx*^*m *^transcript. The amplicons were sequenced for verification. For loading control a separate RT-PCR of each mRNA sample was carried out in parallel using a primer pair (*β3 *sense 5'-ATCATTTCCGAGGAGCACGGC-3'; *β3 *antisense 5'-GCCCAGCGAGTGCGTCAATTG-3') for the ubiquitously expressed gene *β3-tubulin *[48], which amplifies a 397 bp fragment from the transcript.

### Antibodies and immunofluorescence staining

The polyclonal DSX^M ^antibody was made using standard methods (Covance, Princeton, NJ). A peptide containing the C-terminal amino acids SSNGAYHHGHHL was used for immunization of two rats, both of which gave useful antibodies of good titer and were used here.

For cell staining experiments, pre-stage 17 embryos were fixed, devitellinized, and immunostained as described [49]. Because the presence of the cuticle greatly hinders antibody staining, we used a different protocol for stage 17. Fixed stage 17 embryos were rehydrated, washed twice in 1 × PBS + 0.1% Tween 20 (PBT), sonicated in 500 μl PBT (2 pulses of 3 sec. using a Misonix Sonicator 3000 at output setting 1), rewashed twice in PBT and immunostained [49]. Gonads of larvae and adult flies were dissected, fixed and immunostained as described [50]. All stained samples were mounted on slides in Fluoromount-G (Southern Biotech, Birmingham, AL).

Antibodies were as follows: mouse anti-FASIII and mouse anti-EYA10H6 (Developmental Studies Hybridoma Bank, Iowa City, IA) at 1:50, rabbit anti-SOX100B [28] at 1:1000, guinea pig anti-TJ [26] at 1:3000, rabbit anti-VASA (R. Lehmann) at 1:5000, chicken anti-VASA [51] at 1:1000. For the rat anti-DSX^M ^at 1:500 in general and the rabbit anti-SOX100B at 1:1000 (in Fig 4N) the TSA (Tyramide Signal Amplification) Cyanine 3 technology (Perkin Elmer, Waltham, MA) in combination with the ABC Kit (Vector Laboratories, Burlingham, CA) was used as the detection system.

The following secondary antibodies were used, all at 1:500: Cy5 goat anti-rabbit, biotin-coupled goat anti-rat, and biotin-coupled goat anti-rabbit (Jackson Immuo-research, West Grove, PA), Alexa 647 goat anti-guinea pig, Alexa 488 goat anti-guinea pig, Alexa 488 goat anti-rabbit, Alexa 647 goat anti-mouse, Alexa 488 goat anti-mouse, Alexa 647 goat anti-chicken (Invitrogen Molecular Probes, Carlsbad, CA). Images were acquired using a Zeiss LSM 510 Meta Confocal Microscope (Zeiss, Thornwood, NY) and processed using Photoshop 7.0 (Adobe, San Jose, CA). All figures were annotated using Illustrator 10.0 (Adobe, San Jose, CA). All images in figures are single optical sections as indicated.

DAPI (4',6-diamidino-2-phenylindole, dihydrochloride) staining was used to visualize the nuclei.

## Supplementary Material

Additional file 1 DSX^M ^localizes to the nucleus. (A-D) Stage 13 testis immunofluorescence using (A) anti- DSX^M^, (B) anti-VASA, and (C) DAPI. (D) Merged images A-C. (E-H) Stage 15 male testis immunofluorescence using (E) anti- DSX^M^, (F) anti-VASA and (G) DAPI. (H) Merged images E-G. The scale bars = 10 mm. Anterior is to the left. Click here for file
