# Supplementary figures and images for: Sex-specific DoublesexM expression in subsets of Drosophila somatic gonad cells
Source: BMC Dev Biol. 2007 Oct 12;7:113. doi: 10.1186/1471-213X-7-113 (PMC2148063; doi:10.1186/1471-213X-7-113)

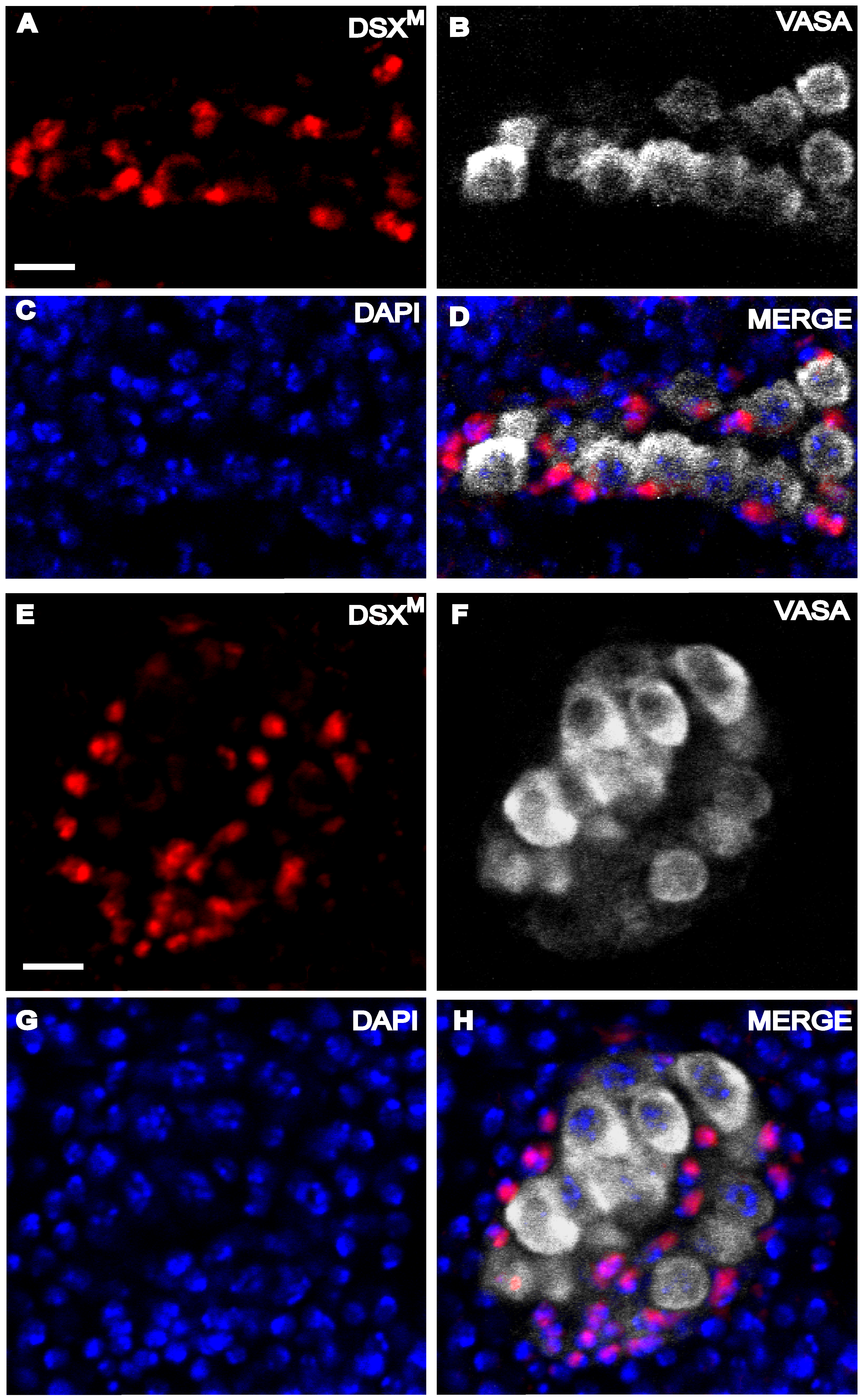

Supplement: Additional file 1 — DSXM localizes to the nucleus. (A-D) Stage 13 testis immunofluorescence using (A) anti- DSXM, (B) anti-VASA, and (C) DAPI. (D) Merged images A-C. (E-H) Stage 15 male testis immunofluorescence using (E) anti- DSXM, (F) anti-VASA and (G) DAPI. (H) Merged images E-G. The scale bars = 10 mm. Anterior is to the left. [file 1471-213X-7-113-S1.tiff]
